# Difficulties in interpreting patient-reported outcome measures in the absence of a gold standard: the meaning of Clinical Global Impressions scores in liaison psychiatry

**DOI:** 10.1192/bjb.2021.47

**Published:** 2021-06

**Authors:** Rajalakshmi Valaiyapathi, Kezia Smith, Ksenia Marjanovic-Deverill

**Affiliations:** West London NHS Trust, UK. Email: r.valaiyapathi@nhs.net; West London NHS Trust, UK. Email: kezia.smith2@nhs.net; Liaison Psychiatry Consultant, West London NHS Trust, UK. Email: ksenia.marjanovic-deverill@westlondon.nhs.uk

As a part of routine clinical care, the Ealing Liaison Psychiatry Service (ELPS) uses the Clinical Global Impressions (CGI) scale to assess patient-reported outcomes. We would like to share our findings and the challenges in interpreting CGI ratings.

CGI scales in psychiatry were initially used to assess efficacy in clinical drug trials^1^ and have since been adapted for use in liaison psychiatry as a part of a nationwide evaluation. The Framework for Routine Outcome Measurement in Liaison Psychiatry^2^ proposed that all liaison psychiatry services use CGI scales for consistent data collection and national reporting of outcomes, although there is no guidance on a standard to aim for.

Our methodology involved patients and their ELPS clinicians providing a CGI rating on whether the patient's mental health had improved, not changed or become worse after ELPS contact. This study looked at all 205 patients between January 2018 and November 2019 who had filled out a CGI questionnaire, and the following analyses were made:

- percentages of patients reporting changes in their mental health and potential reasons for this;
- concordance between patient and clinician ratings.

Fifty-nine per cent of patients reported an improvement in their mental health, although the reasons for this were unclear, given that the CGI questionnaire has no section for patients to justify their rating. A variety of factors may be involved, including having a focused consultation with a clinician, a decrease in symptom severity, and improvements in physical or social symptoms during the hospital stay, as these are often inextricably linked with mental health.

Forty per cent felt there was no change after ELPS intervention, and 1% (three patients) indicated feeling worse. Of the latter, two patients had to be admitted to an in-patient psychiatric unit, which could suggest that their lack of improvement was due to the severity of their mental health condition itself. There was 91% concordance between patient and clinician ratings, suggesting that subjective ratings from patients may not be needed if clinicians’ objective ratings are so closely tallied.

The CGI scale has been correlated with more time-consuming rating scales used in psychiatry,^3^ and its advantages lie in its ease of understanding by both professionals and lay people, its ability to track progress across time and its swiftness of application. On reflection of our findings, we are unable to comment on our performance given the dearth of literature discussing what constitutes the gold standard. However, encouragingly, most patients reported improvement while only a very small minority reported deterioration, indicating that liaison psychiatry interventions are effective and largely beneficial.

By nature, the liaison psychiatry population comprises patients with both physical and mental health conditions, causing relative difficulty in teasing out which of the two issues is better manifested in the desirable outcome. In addition, the heterogeneity of the liaison psychiatry population makes it difficult to make direct comparisons of validity between different psychiatric conditions.

This simple service evaluation suggests that ELPS improves patients’ well-being according to CGI scales. Nevertheless, wider-scale studies should be performed to elucidate how liaison psychiatry interventions are beneficial and to inform what the standard of care should be.
